# Supplementary material for: Epigenetic memory is governed by an effector recruitment specificity toggle in Heterochromatin Protein 1
Source: Nat Commun. 2024 Jul 25;15:6276. doi: 10.1038/s41467-024-50538-z (PMC11272775; doi:10.1038/s41467-024-50538-z)
Supplement: Supplementary file 3 — Description of Additional Supplementary Files [file 41467_2024_50538_MOESM3_ESM.pdf]

Description of Additional Supplemental Files

File Name: Supplementary Data 1
Description: TMT-MS peptide results dataset

File Name: Supplementary Data 2
Description: *S. pombe* strains used in this study.

File Name: Supplementary Data 3
Description: Oligos used in this study.
