# Supplementary material for: Epigenetic memory is governed by an effector recruitment specificity toggle in Heterochromatin Protein 1
Source: Nat Commun. 2024 Jul 25;15:6276. doi: 10.1038/s41467-024-50538-z (PMC11272775; doi:10.1038/s41467-024-50538-z)
Supplement: Supplementary file 5 — Supplementary Data 2 [file 41467_2024_50538_MOESM5_ESM.pdf]

Supplementary Data 2*. S.pombe* strains used in this study.

Strain no.	Strain genotype	Source	Related to
KR18	*h- leu1-32 ade6-M210 ura4Δ::10XtetO-ade6 clr4+ trp1:nat-clr4p-tetR-clr4ΔCD*	Moazed Lab	Figures 1B-G, 2A-B,2E, 3A-B, 5F Supplementary Figures 1B-D, 2, 3D, 11A-B
KR24	*h- leu1-32 ade6-M210 ura4Δ::10XtetO-ade6, clr4Δ::kanMX6*	Moazed Lab	Figures 1B-C,1E-G, 2A-B,2E, 3A-B Supplementary Figures 1D, 2, 3D
KR33	*h- leu1-32 ade6-M210 ura4Δ::10XtetO-ade6 clr4+ trp1:nat-clr4p-tetR-clr4ΔCD epe1Δ::kanMX6*	Moazed Lab	Figures 1B-G, 2A-B,E, 3A-B, 5E Supplementary Figures 1D, 2, 3D, 11A-B
KR283	*h- leu1-32 ade6? ura4Δ::10XtetO-ade6 clr4+, trp1:nat-clr4p-tetR-clr4ΔCD , epe1Δ::3XFLAG-epe1-hph*	Moazed Lab	Supplementary Figure 3F
KR343	*h+ otr1R(SphI)::ade6+ ura4-D18 leu1-32 ade6-M210*	Moazed Lab	Figures 2C-D, 3D, 5A-B, Supplementary Figure 9A
KR453	*h+ otr1R(SphI)::ade6+ ura4-D18 leu1-32 ade6-M210 swi6D::ura4 3XFLAG-Swi6*	Moazed Lab	Figure 5A-C Supplementary Figure 9A
KR555	*h+ otr1R(SphI)::ade6+ ura4-D18 leu1-32 ade6-M210, clr4D::ura4-kanMX6*	Moazed Lab	Figure 2C-D
KR778	*h90 leu1-32 ade6M216 ura4D-18 swi6: PAmCherry-Swi6*	This study	Figure 4E-G
KR1101	*h- leu1-32 ade6-M210 tandem ura4Δ::10XUAS-10XtetO-ade6 natMX6-tetR-clr4-I epe1+ leu1:nmt1-Gal4-Clr3 swi6Δ::*	This study	Supplementary Figure 11A
KR1124	*h+ leu1-32 ura4Δ::10XUAS-tetO-ade6+ natMX6-tetR-clr4-I epe1+ leu1:nmt1-gal4-clr3*	This study	Supplementary Figure 11A
KR1982	*h- leu1-32 ade6-M210 ura4Δ::10XtetO-ade6 clr4+ trp1:nat-clr4p-tetR-clr4ΔCD swi6T278S*	This study	Figure 1B Supplementary Figure 1B
KR1984	*h- leu1-32 ade6-M210 ura4Δ::10XtetO-ade6 clr4+ trp1:nat-clr4p-tetR-clr4ΔCD swi6T278Y*	This study	Figure 1B,1D-G, 2A-B,E, 3A-B, 5E Supplementary Figure 1B-C
KR1986	*h- leu1-32 ade6-M210 ura4Δ::10XtetO-ade6 clr4+ trp1:nat-clr4p-tetR-clr4ΔCD swi6T278F*	This study	Figure 1B Supplementary Figure 1B
KR1988	*h- leu1-32 ade6-M210 ura4Δ::10XtetO-ade6 clr4+ trp1:nat-clr4p-tetR-clr4ΔCD swi6T278C*	This study	Figure 1B Supplementary Figure 1B
KR1990	*h- leu1-32 ade6-M210 ura4Δ::10XtetO-ade6 clr4+ trp1:nat-clr4p-tetR-clr4ΔCD swi6T278K*	This study	Figure 1C-F, 2A-B, 2E, 3A-B Supplementary Figure 1B-C, 11A-B

KR1994	*h- leu1-32 ade6-M210 ura4∆::10XtetO-ade6 clr4+ trp1:nat-clr4p-tetR-clr4∆CD epe1-V5-kanMX6*	This study	Figure 3D
KR1997	*h- leu1-32 ade6-M210 ura4∆::10XtetO-ade6 clr4+ trp1:nat-clr4p-tetR-clr4∆CD swi6T278Y epe1∆::kanMX6*	This study	Figure 1F, 3A-B Supplementary Figure 1D
KR1999	*h- leu1-32 ade6-M210 ura4∆::10XtetO-ade6 clr4+ trp1:nat-clr4p-tetR-clr4∆CD ckb1D::kanMX6*	This study	Figure 1G
KR2000	*h- leu1-32 ade6-M210 ura4∆::10XtetO-ade6 clr4+ trp1:nat-clr4p-tetR-clr4∆CD swi6T278Y ckb1∆::kanMX6*	This study	Figure 1G
KR2003	*h+ otr1R(SphI)::ade6+ ura4-D18 leu1-32 ade6-M210 swi6∆::ura4 3XFLAG-swi6T278Y*	This study	Figure 5A, C Supplementary Figure 9A
KR2009	*h+ otr1R(SphI)::ade6+ ura4-D18 leu1-32 ade6-M210 swi6T278Y*	This study	Figure 2C-D
KR2041	*h+ otr1R(SphI)::ade6+ ura4-D18 leu1-32 ade6-M210 swi6T278K*	This study	Figure 2C-D
KR2101	*h- leu1-32 ade6-M210 ura4∆::10XtetO-ade6 clr4+ trp1:nat-clr4p-tetR-clr4∆CD swi6∆270-328::ura4-hphMX6*	This study	Supplementary Figure 1A
KR2436	*h- leu1-32 ade6-M210 ura4∆::10XtetO-ade6 clr4+ trp1:nat-clr4p-tetR-clr4∆CD crb3-TAP-kanMX6*	This study	Figure 5F
KR2441	*h- leu1-32 ade6-M210 ura4∆::10XtetO-ade6 clr4+ trp1:nat-clr4p-tetR-clr4D∆CD epe1-V5-hphMX6 swi6T278Y*	This study	Figure 3D
KR2443	*h- leu1-32 ade6-M210 ura4∆::10XtetO-ade6 clr4+ trp1:nat-clr4p-tetR-clr4∆CD epe1-V5-hphMX6 swi6T278K*	This study	Figure 3D
KR2445	*h90 leu1-32 ade6M216 ura4D-18 swi6: PAmCherry-swi6T278K*	This study	Figure 4F-G
KR2465	*h- leu1-32 ade6-M210 ura4∆::10XtetO-ade6 trp1:nat-clr4p-tetR-clr4∆CD swi6T278Y grc3V70M*	This study	Figure 5E
KR2468	*h- leu1-32 ade6-M210 ura4∆::10XtetO-ade6 trp1:nat-clr4p-tetR-clr4∆CD epe1∆-kanMX6 grc3V70M*	This study	Figure 5E
KR2471	*h90 leu1-32 ade6M216 ura4D-18 swi6: PAmCherry-swi6T278Y*	This study	Figure 4E, G

KR2474	*h- leu1-32 ade6-M210 ura4Δ::10XtetO-ade6 clr4+ trp1:nat-clr4p-tetR-clr4ΔCD swi6T278Y crb3-TAP-kanMX6*	This study	Figure 5F
KR2650	*h- leu1-32 ade6-M210 ura4Δ::10XtetO-ade6 clr4+ trp1:nat-clr4p-tetR-clr4ΔCD swi6T278K epe1Δ::kanMX6*	This study	Figure 1F, 3A-B Supplementary Figure 1D
KR2686	*h- leu1-32 ade6-M210 ura4Δ::10XUAS-tetO-ade6 natMX6-tetR-clr4-I epe1+ leu1:nmt1-gal4-clr3 swi6T278K*	This study	Supplementary Figure 11A
KR2700	*h- leu1-32 ade6-M210 ura4Δ::10XtetO-ade6 clr4+ trp1:nat-clr4p-tetR-clr4ΔCD swi6T278K crb3-TAP-kanMX6*	This study	Figure 5F
KR2703	*h- leu1-32 ade6-M210 ura4Δ::10XtetO-ade6 clr4+ trp1:nat-clr4p-tetR-clr4ΔCD grc3V70M crb3-TAP-kanMX6*	This study	Figure 5F
KR2736	*h- leu1-32 ade6-M210 ura4Δ::10XtetO-ade6 clr4+ trp1:nat-clr4p-tetR-clr4ΔCD swi6T278R*	This study	Figure 1C Supplementary Figure 1B
KR2798	*h- leu1-32 ade6-M210 ura4Δ::10XtetO-ade6 clr4+ trp1:nat-clr4p-tetR-clr4ΔCD swi6T278A*	This study	Figure 1B Supplementary Figure 1B
KR2799	*h- leu1-32 ade6-M210 ura4Δ::10XtetO-ade6 clr4+ trp1:nat-clr4p-tetR-clr4ΔCD swi6T278D*	This study	Figure 1C Supplementary Figure 1B
KR2801	*h- leu1-32 ade6-M210 ura4Δ::10XtetO-ade6 clr4+ trp1:nat-clr4p-tetR-clr4ΔCD swi6T278E*	This study	Figure 1C Supplementary Figure 1B
KR2825	*h+ otr1R(SphI)::ade6+ ura4-D18 leu1-32 ade6-M210 swi6Δ::ura4 3XFLAG-swi6T278K*	This study	Figure 5B-C Supplementary Figure 9A
KR2827	*h- leu1-32 ade6-M210 ura4Δ::10XtetO-ade6 clr4+ trp1:nat-clr4p-tetR-clr4ΔCD ckb1Δ::kanMX6 epe1Δ::hphMX6*	This study	Figure 1G
KR2867	*h+ leu1-32 ura4Δ::10XUAS-tetO-ade6 natMX6-tetR-clr4-I epe1+ leu1:nmt1-gal4-clr3 grc3V70M*	This study	Supplementary Figure 11A
KR3086	*h- leu1-32 ade6? ura4Δ::10XtetO-ade6 clr4+, trp1:nat-clr4p-tetR-clr4ΔCD , epe1Δ::3XFLAG-epe1Δ566-600-hph* #1	This study	Supplementary Figure 3D,F
KR3087	*h- leu1-32 ade6? ura4Δ::10XtetO-ade6 clr4+, trp1:nat-clr4p-tetR-clr4ΔCD , epe1Δ::3XFLAG-epe1Δ566-600-hph* #2	This study	Supplementary Figure 3D

KR3127	*h- leu1-32 ade6? ura4Δ::10XtetO-ade6 clr4+, trp1:nat-clr4p-tetR-clr4ΔCD , epe1Δ::3XFLAG-epe1Δ569-573-hph*	This study	Supplementary Figure 3D,F
KR3167	*h- leu1-32 ade6? ura4Δ::10XtetO-ade6 clr4+, trp1:nat-clr4p-tetR-clr4ΔCD , epe1Δ::3XFLAG-epe1Δ577-589-hph*	This study	Supplementary Figure 3D,F
KR3284	*h- leu1-32 ade6-M210 ura4Δ::10XtetO-ade6 clr4+ trp1:nat-clr4p-tetR-clr4ΔCD swi6T278KD283H*	This study	Supplementary Figure 11B
KR3285	*h- leu1-32 ade6-M210 ura4Δ::10XtetO-ade6 clr4+ trp1:nat-clr4p-tetR-clr4ΔCD swi6T278KD283T*	This study	Supplementary Figure 11B
KR3286	*h- leu1-32 ade6-M210 ura4Δ::10XtetO-ade6 clr4+ trp1:nat-clr4p-tetR-clr4ΔCD swi6T278KD283S*	This study	Supplementary Figure 11B
KR3287	*h- leu1-32 ade6-M210 ura4Δ::10XtetO-ade6 clr4+ trp1:nat-clr4p-tetR-clr4ΔCD swi6T278KD283R*	This study	Supplementary Figure 11B
KR3288	*h- leu1-32 ade6-M210 ura4Δ::10XtetO-ade6 clr4+ trp1:nat-clr4p-tetR-clr4ΔCD swi6T278KD283E*	This study	Supplementary Figure 11B
