# Supplementary material for: Epigenetic memory is governed by an effector recruitment specificity toggle in Heterochromatin Protein 1
Source: Nat Commun. 2024 Jul 25;15:6276. doi: 10.1038/s41467-024-50538-z (PMC11272775; doi:10.1038/s41467-024-50538-z)
Supplement: Supplementary file 7 — Reporting Summary [file 41467_2024_50538_MOESM7_ESM.pdf]

nature portfolio

Corresponding author(s): Kaushik Ragunathan

Last updated by author(s): Jun 9, 2024

Reporting Summary

Nature Portfolio wishes to improve the reproducibility of the work that we publish. This form provides structure for consistency and transparency in reporting. For further information on Nature Portfolio policies, see our Editorial Policies and the Editorial Policy Checklist.

Statistics

For all statistical analyses, confirm that the following items are present in the figure legend, table legend, main text, or Methods section.

n/a	Confirmed	
☐	☒	The exact sample size (*n*) for each experimental group/condition, given as a discrete number and unit of measurement
☐	☒	A statement on whether measurements were taken from distinct samples or whether the same sample was measured repeatedly
☐	☒	The statistical test(s) used AND whether they are one- or two-sided<br>*Only common tests should be described solely by name; describe more complex techniques in the Methods section.*
☒	☐	A description of all covariates tested
☒	☐	A description of any assumptions or corrections, such as tests of normality and adjustment for multiple comparisons
☐	☒	A full description of the statistical parameters including central tendency (e.g. means) or other basic estimates (e.g. regression coefficient) AND variation (e.g. standard deviation) or associated estimates of uncertainty (e.g. confidence intervals)
☐	☒	For null hypothesis testing, the test statistic (e.g. *F*, *t*, *r*) with confidence intervals, effect sizes, degrees of freedom and *P* value noted<br>*Give P values as exact values whenever suitable.*
☐	☒	For Bayesian analysis, information on the choice of priors and Markov chain Monte Carlo settings
☒	☐	For hierarchical and complex designs, identification of the appropriate level for tests and full reporting of outcomes
☒	☐	Estimates of effect sizes (e.g. Cohen's *d*, Pearson's *r*), indicating how they were calculated

Our web collection on statistics for biologists contains articles on many of the points above.

Software and code

Policy information about availability of computer code

Data collection	AlphaFold2 Multimer, Refeyn AcquireMP, https://github.com/BiteenMatlab/SMALL-LABS, BioRAD CFX Maestro
Data analysis	ChimeraX-1.6.1, Prism 10, ImageJ, IGV_2.16.12, ggplot, pheatmap, https://github.com/sokrypton/ColabFold/tree/main/colabfold, Refeyn DiscoverMP, barcode, Trimmomatic, BWA, https://github.com/BiteenMatlab/SMAUG, RColorBrewer, SummarizedExperiment, matrixStats, tidyverse

For manuscripts utilizing custom algorithms or software that are central to the research but not yet described in published literature, software must be made available to editors and reviewers. We strongly encourage code deposition in a community repository (e.g. GitHub). See the Nature Portfolio guidelines for submitting code & software for further information.

Data

Policy information about availability of data

All manuscripts must include a data availability statement. This statement should provide the following information, where applicable:

- Accession codes, unique identifiers, or web links for publicly available datasets
- A description of any restrictions on data availability
- For clinical datasets or third party data, please ensure that the statement adheres to our policy

The sequencing data generated in this study have been deposited in NCBI's Gene Expression Omnibus (GEO) and are accessible through GEO Series accession number GSE248428 [https://www.ncbi.nlm.nih.gov/geo/query/acc.cgi?acc= GSE248428]. The mass spectrometry proteomics data generated in this study have

been deposited to the ProteomeXchange Consortium via the PRIDE partner repository with the dataset identifier PXD047651 [http://proteomecentral.proteomexchange.org/cgi/GetDataset?ID= PXD047651] 69,70. Source data are provided with this paper. The Alphafold-2 multimer models are available in ModelArchive (www.modelarchive.org) with the accession codes ma-dl26b, ma-n4bjg, ma-01x2c, and ma-j3lwp.

Research involving human participants, their data, or biological material

Policy information about studies with human participants or human data. See also policy information about sex, gender (identity/presentation), and sexual orientation and race, ethnicity and racism.

Reporting on sex and gender	Human participants, their data, or their biological material were not involved in this research study.
Reporting on race, ethnicity, or other socially relevant groupings	Human participants, their data, or their biological material were not involved in this research study.
Population characteristics	Human participants, their data, or their biological material were not involved in this research study.
Recruitment	Human participants, their data, or their biological material were not involved in this research study.
Ethics oversight	Human participants, their data, or their biological material were not involved in this research study.

Note that full information on the approval of the study protocol must also be provided in the manuscript.

Field-specific reporting

Please select the one below that is the best fit for your research. If you are not sure, read the appropriate sections before making your selection.

☒ Life sciences ☐ Behavioural & social sciences ☐ Ecological, evolutionary & environmental sciences

For a reference copy of the document with all sections, see nature.com/documents/nr-reporting-summary-flat.pdf

Life sciences study design

All studies must disclose on these points even when the disclosure is negative.

Sample size	Sample sizes were chosen based on what is reported in literature for similar studies. Each sample size was determined with the rationale of maximizing confidence and reproducibility of our findings.
Data exclusions	No data was excluded from this study.
Replication	We verified reproducibility for all of our experiments by including technical or biological replicates. Experiments using recombinant protein (binding assays, mass photometry) were performed with a minimum of three technical replicates. CoIP, ChIP, and purified-RNA experiments were performed with 2 technical replicates, except for the Quantitative AP-MS which was performed with 3 technical replicates and the TAP ChIP which was performed with 3-4 replicates. Most strains reported in this study have at least one biological replicate that reproduces the indicated silencing phenotype. We kept the size of our experimental groups consistent and included necessary controls across replicates.
Randomization	We handled our samples to avoid bias whenever possible. We primarily used simple randomization techniques, including changing sample order between technical replicates to account for any covariates that may arise from experimental design.
Blinding	Blinding is only relevant to preventing data analysis bias in our study. During data collection and analysis, samples were assigned numbers to prevent bias. Additionally, contributing investigators were not made aware of the expected results for experimental variables until after the data was analyzed.

Reporting for specific materials, systems and methods

We require information from authors about some types of materials, experimental systems and methods used in many studies. Here, indicate whether each material, system or method listed is relevant to your study. If you are not sure if a list item applies to your research, read the appropriate section before selecting a response.

Materials & experimental systems

n/a	Involved in the study
☐	☒ Antibodies
☐	☒ Eukaryotic cell lines
☒	☐ Palaeontology and archaeology
☒	☐ Animals and other organisms
☒	☐ Clinical data
☒	☐ Dual use research of concern
☒	☐ Plants

Methods

n/a	Involved in the study
☐	☒ ChIP-seq
☒	☐ Flow cytometry
☒	☐ MRI-based neuroimaging

Antibodies

Antibodies used	V5 (A01724, Genscript, 1:10,000 dilution)<br>Swi6 (custom, courtesy of Moazed Lab, Department of Cell Biology, Harvard Medical School, 1:10,000 dilution)<br>MBP (E8032S, NEB, 1:10,000 dilution)<br>M2 Flag (A8592, Sigma, 1:10,000 dilution)<br>H3K9me2 (Abcam, ab1220)<br>H3K9me3 (39161, Active Motif)<br>IgG Alexa Fluor 647 (A-21246, Invitrogen, 1:10,000 dilution)
Validation	All antibodies were commercially validated and experimentally validated using proper controls. For all experiments in the manuscript, positive and negative controls were included to verify the specificity of each antibody.

Eukaryotic cell lines

Policy information about cell lines and Sex and Gender in Research

Cell line source(s)	We used S. pombe lab strains, strains provided upon request.
Authentication	S. pombe strains were authenticated using PCR-based genotyping.
Mycoplasma contamination	S. pombe strains do not need to be tested for mycoplasma contamination.
Commonly misidentified lines<br>(See ICLAC register)	This does not apply to S. pombe.

Plants

Seed stocks	Plants were not used in this study.
Novel plant genotypes	Plants were not used in this study.
Authentication	Plants were not used in this study.

ChIP-seq

Data deposition

☒ Confirm that both raw and final processed data have been deposited in a public database such as GEO.

☒ Confirm that you have deposited or provided access to graph files (e.g. BED files) for the called peaks.

Data access links<br>*May remain private before publication.*	https://www.ncbi.nlm.nih.gov/geo/query/acc.cgi?acc=GSE248428
Files in database submission	GSM7913848 WT_9me2_minustet<br>GSM7913849 clr4D_9me2_minustet<br>GSM7913850 epe1D_9me2_minustet<br>GSM7913851 swi6T278Y_9me2_minustet

GSM7913852 swi6T278K_9me2_minustet
GSM7913853 swi6T278Yepe1D_9me2_minustet
GSM7913854 swi6T278Kepe1D_9me2_minustet
GSM7913855 WT_9me2_tet
GSM7913856 clr4D_9me2_tet
GSM7913857 epe1D_9me2_tet
GSM7913858 swi6T278Y_9me2_tet
GSM7913859 swi6T278K_9me2_tet
GSM7913860 swi6T278Yepe1D_9me2_tet
GSM7913861 swi6T278KYepe1D_9me2_tet
GSM7913862 WT_9me3_minustet
GSM7913863 clr4D_9me3_minustet
GSM7913864 epe1D_9me3_minustet
GSM7913865 swi6T278Y_9me3_minustet
GSM7913866 swi6T278K_9me3_minustet
GSM7913867 swi6T278Yepe1D_9me3_minustet
GSM7913868 swi6T278Kepe1D_9me3_minustet
GSM7913869 WT_9me3_tet
GSM7913870 clr4D_9me3_tet
GSM7913871 epe1D_9me3_tet
GSM7913872 swi6T278Y_9me3_tet
GSM7913873 swi6T278K_9me3_tet
GSM7913874 swi6T278YepeD_9me3_tet
GSM7913875 swi6T278Kepe1D_9me3_tet
GSE248428_RAW.tar
GSE248428_Schizosaccharomyces_Pombe_tetarr6.fasta.gz
GSE248428_Schizosaccharomyces_Pombe_tetarr6.gff.gz

Genome browser session (e.g. UCSC)

We did not use a genome browser for this study.

Methodology

Replicates

ChIP was performed on two technical replicates, qPCR was performed on both replicates to ensure they were in agreement, and ChIP-seq was performed on one replicate

Sequencing depth

1- 5 million pair-end raw reads for each sample with the length of 75 bp, >98% of our reads map to the S. pombe genome

Antibodies

H3K9me2 (Abcam, ab1220)
H3K9me3 (39161, Active Motif)

Peak calling parameters

Peak calling was not used in ChIP-seq analysis for this study.

Data quality

We did not use peak calling or FDR for this study.

Software

barcode, Trimmomatic, BWA, IGV_2.16.12
